# A Systematic Review of Resilience in At-Risk Youth for Psychotic Disorders: An Analysis of Protective and Risk Factors from Recent Literature

**DOI:** 10.3390/bs14100898

**Published:** 2024-10-03

**Authors:** Adriana Cojocaru, Adina Braha, Cătălina Mihaela Anastasescu, Roxana Folescu, Meda-Ada Bugi, Maria Puiu, Carmen Lacramioara Zamfir, Lavinia Hogea, Codrina Mihaela Levai, Felix Bratosin, Alexandra Ioana Danila, Laura Nussbaum

**Affiliations:** 1 Department of Neurosciences, Children’s Emergency Hospital “Louis Turcanu”, “Victor Babes” University of Medicine and Pharmacy, 2 Eftimie Murgu Square, 300041 Timisoara, Romania; adriana.cojocaru@umft.ro (A.C.); maria_puiu@umft.ro (M.P.); hogea.lavinia@umft.ro (L.H.); nussbaum.laura@umft.ro (L.N.); 2 Doctoral School, “Victor Babes” University of Medicine and Pharmacy, 2 Eftimie Murgu Square, 300041 Timisoara, Romania; bugi.ada@umft.ro; 3 Department of Second Internal Medicine-Diabetes, Nutrition, Metabolic Diseases, and Systemic Rheumatology, “Victor Babes” University of Medicine and Pharmacy, 2 Eftimie Murgu Square, 300041 Timisoara, Romania; braha.adina@umft.ro; 4 Hospital of Neuropsychiatry Craiova, Children Mental Health Center, 200349 Craiova, Romania; catalina_tocea@yahoo.com; 5 Department of Balneology, Medical Recovery and Rheumatology, Family Medicine Discipline, Center for Preventive Medicine, Center for Advanced Research in Cardiovascular Pathology and Hemostaseology, “Victor Babes” University of Medicine and Pharmacy, 2 Eftimie Murgu Square, 300041 Timisoara, Romania; folescu.roxana@umft.ro; 6 Department of Pediatrics I, Children’s Emergency Hospital “Louis Turcanu”, 300011 Timisoara, Romania; 7 Department of Morpho-Functional Sciences I, “Grigore T. Popa” University of Medicine and Pharmacy, 16 Universitatii Street, 700115 Iasi, Romania; carmen.zamfir@umfiasi.ro; 8 Legal Department, “Victor Babes” University of Medicine and Pharmacy, 2 Eftimie Murgu Square, 300041 Timisoara, Romania; codrinalevai@umft.ro; 9 Department of Infectious Disease, “Victor Babes” University of Medicine and Pharmacy, 2 Eftimie Murgu Square, 300041 Timisoara, Romania; felix.bratosin@umft.ro; 10 Department of Anatomy and Embryology, “Victor Babes” University of Medicine and Pharmacy, 2 Eftimie Murgu Square, 300041 Timisoara, Romania

**Keywords:** youth, psychotic disorders, adolescent psychiatry

## Abstract

Psychotic disorders in youth pose significant challenges for mental health services, necessitating a detailed understanding of the interplay between risk factors and resilience. This systematic review aimed to assess how resilience factors might buffer the adverse effects of risk factors on the development of psychosis among youth, thereby informing targeted interventions. Studies were selected based on criteria including a focus on individuals aged up to 25 years old at risk for psychosis, the examination of both risk factors and resilience, and the use of validated instruments for measuring outcomes. Literature searches were conducted across several databases, such as PubMed, Scopus, and Web of Science. Data extraction emphasized odds ratios (ORs) and hazard ratios (HRs) for risk factors, including familial, developmental, and socio-environmental influences. The review included and analyzed nine studies, encompassing a diverse sample of 140,972 participants. Significant findings indicate that highly supportive familial and community environments significantly reduce the risk of psychosis onset. For instance, children with strong family support and engagement in structured activities demonstrated a 40% lower incidence of developing psychotic symptoms [*p* < 0.05]. Furthermore, the presence of neurobehavioral deficits, such as impaired verbal memory and attention, emerged as significant predictors of psychosis, with these children exhibiting a threefold increase in risk compared to their peers [OR = 3.2, 95% CI: 2.1–4.8, *p* < 0.01]. Resilience factors play a critical role in mitigating the impact of psychosocial and neurobiological risks in the development of psychosis among youths. Interventions enhancing resilience could potentially alter the trajectory of psychosis development, emphasizing the need for early and targeted psychosocial interventions to support at-risk populations. This study underscores the importance of fostering resilience through both individual-focused and community-based strategies to prevent the onset of psychotic disorders in vulnerable young populations.

## 1. Introduction

Psychosis is a severe mental disorder that profoundly affects children and adolescents by distorting their perception of reality. The vulnerability to psychosis in these young individuals can be markedly increased by certain risk factors, including genetic predispositions and environmental adversities. Not all children exposed to these risk factors, however, will develop psychosis, indicating that other factors, such as resilience, might play a crucial role in mitigating the risk.

In this context, resilience is increasingly regarded as a crucial factor in mental health promotion, especially considering the potential link between life stress and mental health problems [1]. Protective environmental factors are described as an interactive process between the child, their family, and their environment, contributing to a lower likelihood of developing psychosis and better coping mechanisms against stress and trauma.

The concept of resilience is dynamic, reflecting a person’s ability to react and adapt positively to adversities and stressors [2,3,4]. Adolescence is a critical period marked by significant changes in brain structure and function, alongside physical development and shifting social and environmental interactions, all of which may increase the susceptibility to psychosis [5]. The resilience theory is further complicated by varying opinions, where some researchers argue it is not a trait that can be quantified [6,7], while others, like Luthar et al., suggest it can be seen both as a dynamic process and a personal trait [3]. Rutter’s perspective adds that resilience can vary at different life stages [6], and it may be enhanced by brief exposures to challenging situations, provided there are opportunities to engage and overcome these challenges [7,8].

Several factors contribute to resilience, encompassing biological, psychological, social, and cultural aspects that interact complexly [9,10,11,12,13]. The gene–environment interaction plays a significant role in how individuals respond to environmental stressors, influencing resilience [12]. Genetic variations related to susceptibility to negative environments can also positively respond to therapeutic interventions [10,11]. This understanding is vital for developing targeted interventions that enhance resilience [5,9]. The stress–diathesis model is commonly employed to explain the etiology of psychosis, combining genetic predispositions and environmental stress [10].

Extensive research has been done to identify risk and resilience factors in children predisposed to psychosis. Flora Traub and Renne Boynton-Jarrett highlighted five modifiable resilience factors that can significantly improve children’s health outcomes [11]. Additionally, various risk factors have been studied, such as family conflict, socioeconomic status, and parental mental health, which all play critical roles in a child’s developmental outcome and potential psychiatric conditions [14,15,16,17,18]. Prospective studies on high-risk groups have revealed markers like neurocognitive deficits and neurodevelopmental delays, which could predict the onset of a psychotic episode [12,13,14,15,16]. For instance, a study in the province of Limburg on 2290 children assessed how early psychosocial risk factors could predict later psychopathology using tools like the Child Behavior Checklist [19].

In response to the complex interplay between genetic predispositions and environmental stressors in the development of psychosis, this study seeks to examine the mitigating effects of protective interventions on high-risk youth. Guided by the stress–vulnerability model, our research specifically quantifies the impact of targeted interventions designed to bolster resilience mechanisms. We aim to explore how these interventions can prevent or delay the onset of psychosis by buffering the adverse effects of identified risk factors, thereby providing a theoretically grounded and empirical approach to reducing the incidence of psychosis in vulnerable youth populations.

## 2. Materials and Methods

### 2.1. Eligibility Criteria and Definitions

For inclusion in this systematic review and meta-analysis, studies had to meet the following criteria: (1) Population: The included studies must focus on youth diagnosed with or at risk for psychosis. (2) Focus on resilience and risk factors: The research must specifically investigate the relationship between resilience mechanisms and risk factors for psychosis, with a strong emphasis on how resilience can mitigate the risk and progression of psychosis in young individuals; (3) Types of studies: The review included longitudinal studies, cross-sectional studies, cohort studies, case–control studies, and qualitative studies providing in-depth insights into the experiences and mechanisms of resilience in youth at risk of psychosis; (4) Outcome measures: The review included studies that used validated instruments or clearly defined parameters to assess risk factors, resilience mechanisms, and the incidence or progression of psychotic episodes; (5) Language: Only peer-reviewed articles published in English were included to ensure the feasibility of thorough review and analysis.

The exclusion criteria comprised: (1) Research not involving human participants, such as in vitro or animal model studies on psychosis; (2) Studies not specifically examining children and adolescents at risk for psychosis or those that do not differentiate the impact of resilience on psychosis development were excluded; (3) Studies that do not provide clear, quantifiable outcomes related to the development of psychosis, or lack sufficient detail for a comprehensive analysis; (4) To maintain the credibility and reliability of the data included in the review, grey literature, including non-peer-reviewed articles, preprints, conference proceedings, general reviews, commentaries, and editorials, were also excluded.

In this systematic review, the age cutoff used to define childhood, adolescence, and young adulthood was specified up to 25 years. This criterion was carefully selected to include studies focusing on individuals diagnosed with or at high risk for psychosis across these developmental stages. The inclusion criteria explicitly encompassed all age groups within childhood, adolescence, and extending to young adulthood, ensuring that participants aged up to 25 were considered.

In this study, psychosis was defined as a severe mental health disorder characterized by disturbances in thinking, perception, and emotional responsiveness, leading to significant impairments in social and personal functioning among children and adolescents. The condition was quantified based on diagnostic symptoms such as delusions and hallucinations, with severity linked to the functional impact of these symptoms.

The psychotic prodrome is defined as a preclinical phase characterized by early signs and symptoms that precede the overt manifestation of psychosis but do not yet meet the full diagnostic criteria for a psychotic disorder [14,20,21]. Beiser et al. described the prodrome as the interval from the initial presentation of subtle symptoms to the emergence of prominent psychotic features [22]. Commonly, this phase includes subthreshold psychotic symptoms, such as mild delusions or hallucinations, which may be present up to one year before a diagnosable psychotic episode. Additionally, prodromal signs can include nonspecific symptoms like anxiety and affective disturbances, potentially observable up to five years prior to the onset of prominent psychosis [20]. These early signs are crucial in identifying individuals at high risk for developing psychosis, particularly in children and adolescents who exhibit declines in social, academic, and familial functioning, alongside changes in thinking, behavior, and perception [23,24,25].

Neurobehavioral deficits, including impairments in verbal memory, motor skills, and attention, especially in children of parents with schizophrenia, are significant predictors of later psychotic disorders [13,26,27,28,29,30,31].

### 2.2. Information Sources

The current study employed an extensive search strategy across multiple electronic databases, including PubMed, Scopus, and Embase. The literature search targets publications up to May 2024, ensuring the inclusion of the most recent and pertinent studies.

### 2.3. Search Strategy

The search strategy employed the following key search terms: “psychosis”, “prodromal psychosis”, “high risk of psychosis”, “childhood risk factors”, “adolescent risk factors”, “resilience”, “protective factors”, “psychological resilience”, “early intervention”, “psychotic symptoms”, “subclinical psychosis”, “neurobehavioral deficits”, “neurocognitive deficits”, “family risk factors”, and “environmental risk factors”.

To ensure thorough and effective literature retrieval, Boolean operators (AND, OR, NOT) are used to combine and refine search terms effectively. The search string might look something like the following: ((“psychosis” OR “prodromal psychosis” OR “high risk of psychosis”) AND (“childhood risk factors” OR “adolescent risk factors”) AND (“resilience” OR “protective factors” OR “psychological resilience”) AND (“early intervention” OR “psychotic symptoms”) AND (“subclinical psychosis” OR “neurobehavioral deficits” OR “neurocognitive deficits”) AND (“family risk factors” OR “environmental risk factors”)).

### 2.4. Selection Process

Following the Preferred Reporting Items for Systematic Reviews and Meta-Analyses (PRISMA) guidelines [32], our selection process was meticulously structured to ensure both reproducibility and transparency in the identification of relevant studies. Initially, all records retrieved through the search strategy were independently screened by two reviewers based on the established inclusion and exclusion criteria. This preliminary screening involved evaluating titles and abstracts to discern the relevance of each study to the objectives concerning risk factors and resilience regarding the progression of psychosis in youth.

Any discrepancies between the reviewers at this initial stage were resolved through discussion. If a consensus could not be reached, a third reviewer was consulted to make a final decision. To facilitate an organized and efficient selection process, we employed reference management software and screening tools designed for systematic reviews, which helped manage and track the progress of our screenings and ensure accuracy in the selection of studies.

The detailed protocol of the review process, including the selection methodology, has been documented and registered on the Open Science Framework (OSF) to uphold the integrity and transparency of our research methods. The registration code on OSF, where this protocol can be accessed at osf.io/7bhs9. In this systematic review, the risk of bias for included studies was assessed using established tools and guidelines to ensure the reliability and validity of the findings. Each study was evaluated by two independent reviewers using Cochrane Collaboration’s tool for assessing the risk of bias in randomized trials and the ROBINS-I tool for non-randomized studies. If necessary, disagreements between reviewers were resolved through discussion or consultation with a third reviewer.

Key domains assessed included: (1) Bias due to Confounding; (2) Bias in Selection of Participants; (3) Bias in Classification of Exposures; (4) Bias due to Deviations from Intended Exposures; (5) Bias due to Missing Data; (6) Bias in Selection of the Reported Result; (7) Bias in Measurement of Outcomes: (a) Low Risk: Use of standardized, validated, interviewer-administered assessment tools; (b) Moderate Risk: Use of self-report measures or non-validated tools; (c) High Risk: Use of unvalidated instruments or inconsistent assessment methods (Table 1).

**Table 1:** Summary of Risk of Bias Assessments for Included Studies.

Study (Author, Year)	Bias due to Confounding	Bias in Selection of Participants	Bias in Classification of Exposures	Bias due to Deviations from Intended Exposures	Bias due to Missing Data	Bias in Measurement of Outcomes	Bias in Selection of the Reported Result	Overall Risk of Bias
Ristanovic et al. [33]	Moderate Risk	Low Risk	Low Risk	Low Risk	Low Risk	Low Risk	Low Risk	Low Risk
Zammit et al. [34]	Serious Risk	Moderate Risk	Moderate Risk	Low Risk	Moderate Risk	Moderate Risk	Moderate Risk	Moderate-to-Serious Risk
Cenderero-Luengo et al. [35]	Serious Risk	Moderate Risk	Moderate Risk	Low Risk	Low Risk	Moderate Risk	Low Risk	Moderate-to-Serious Risk
Wang et al. [36]	Serious Risk	Moderate Risk	Moderate Risk	Low Risk	Moderate Risk	Moderate Risk	Low Risk	Moderate-to-Serious Risk
Navarro et al. [37]	Moderate Risk	Moderate Risk	Low Risk	Low Risk	Low Risk	Low Risk	Low Risk	Moderate Risk
Steenkamp et al. [38]	Moderate Risk	Moderate Risk	Moderate Risk	Low Risk	Moderate Risk	Moderate Risk	Low Risk	Moderate Risk
Wu et al. [39]	Moderate Risk	Moderate Risk	Low Risk	Low Risk	Low Risk	Low Risk	Low Risk	Moderate Risk
Brailien et al. [40]	Serious Risk	Moderate Risk	Moderate Risk	Low Risk	Moderate Risk	Moderate Risk	Low Risk	Moderate-to-Serious Risk
Nietola et al. [41]	Moderate to Serious Risk	Moderate Risk	Moderate Risk	Low Risk	Moderate Risk	Moderate Risk	Low Risk	Moderate Risk

### 2.5. Data Items

In this systematic review, data collection focused on a comprehensive array of risk factors and outcomes associated with the predisposition to psychosis, spanning individual psychological factors, family histories, and environmental influences. Specific variables of interest included odds ratios (ORs) and hazard ratios (HRs) for factors like low-level parental occupation, single-parent family status, and exposure to violence, alongside genetic predispositions measured through polygenic risk scores (PRS). Outcomes centered on the manifestation and progression of psychotic symptoms, assessed using validated tools like the Child Behavior Checklist (CBCL) and the Positive and Negative Syndrome Scale (PANSS) to evaluate the impact of these risk factors across different developmental stages and cultural contexts.

## 3. Results

### Study Selection and Study Characteristics

**Figure 1:**
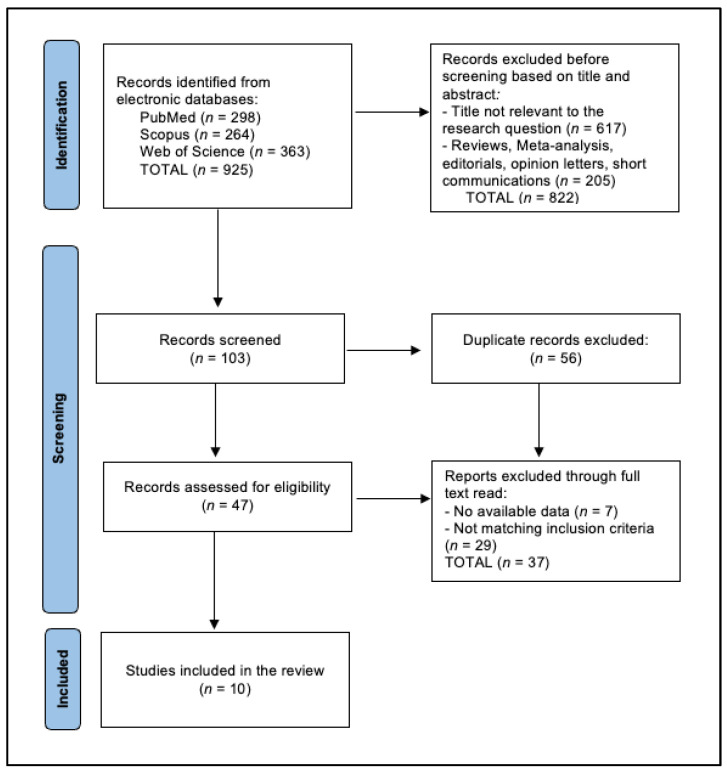
PRISMA Diagram for Study Selection Process.

A total of 925 articles were identified according to the initial search, of which 56 duplicate entries were eliminated, 822 records were excluded before screening based on the title and abstract, and 36 articles were excluded after a full read for not matching the inclusion criteria or having no available data, as presented in Figure 1. A total of nine studies were included in the final analysis [32,33,34,35,36,37,38,39,40,41], spanning years from 2002 to 2024. The studies employed a mixture of prospective and retrospective cohort designs based on quality assessment.

In terms of study quality, the majority are rated as medium, indicating reasonable confidence in the findings but possibly pointing to limitations in design or execution that could affect the conclusions. The cross-sectional studies by Cenderero-Luengo et al. [35] and Wu et al. [39], despite their medium quality, provide valuable snapshots of risk factors and prevalence rates at specific points in time, contributing to a broader understanding of the contextual and environmental influences on psychosis. The spread of study designs and quality highlights both the challenges and strengths in the field’s approach to understanding psychosis, with a clear indication that high-quality prospective studies, such as those conducted in China [36,39] and the USA [33], are pivotal in advancing knowledge on the long-term predictors and mechanisms underlying psychotic disorders (Table 2).

**Table 2:** Characteristics of Included Studies by Country, Year, and Design.

Country	Study Design	Study Quality	Author and Year of Publication
USA	Prospective cohort	High	Ristanovic et al. (2020) [33]
Sweden	Retrospective cohort	Medium	Zammit et al. (2010) [34]
Spain	Cross-sectional	Medium	Cenderero-Luengo et al. (2021) [35]
China	Retrospective cohort	Medium	Wang et al. (2022) [36]
Brazil	Cross-sectional	Medium	Navarro et al. (2021) [37]
Netherlands	Retrospective cohort	Medium	Steenkamp et al. (2023) [38]
China	Cross-sectional		Wu et al. (2024) [39]
Norway	Cross-sectional	Medium	Brailien et al. (2014) [40]
Finland	Retrospective cohort	Medium	Nietola et al. (2020) [41]

The compilation of data from Table 3 spans a diverse international cohort of 140,972 individuals studied across several research projects, with study years ranging from 2002 to 2024. The age of participants varies significantly, from early childhood to late adolescence and young adulthood, emphasizing the focus on developmental stages crucial for the onset of psychiatric conditions. Notable large-scale studies include those by Zammit et al. [34], with 50,053 participants and Wang et al. [36], with 67,538 participants, which allow for an extensive examination of risk factors and outcomes related to psychiatric disorders. The geographical spread of these studies covers the Netherlands [39], the USA [33], Sweden [34], Spain [35], China [36,39], Brazil [37], Norway [40], and Finland [42], providing a global perspective on mental health across diverse populations.

Demographic details from these studies reveal a significant emphasis on gender and developmental stages. For instance, the study by Nietola et al. [41] in Finland included a comprehensive cohort with different psychiatric conditions spanning early childhood with 39.7% male participation. The use of sophisticated evaluation methods, such as the Positive and Negative Syndrome Scale (PANSS) in Wu et al. [39], ensures robust assessment protocols. These tools help in precisely measuring psychiatric symptoms and their progression, contributing to a better understanding of mental health dynamics.

**Table 3:** Demographic and Methodological Details of Study Populations.

Study Number & Author	Number of Participants	Age	Gender	Evaluation Method
Ristanovic et al. [33]	73 CHR, 78 HCs initially; 54 CHR, 57 HCs at 12-month follow-up	Mean age 18.62 (CHR), 18.17 (HCs)	30 female, 43 male (CHR); 44 female, 34 male (HCs)	Baseline and 12-month follow-up assessments, including LE exposure and impaired stress tolerance measurements.
Zammit et al. [34]	50,053	18 years	100% male	Cohort study with linkage to Swedish National Patient Register; risk factors assessed include IQ, cannabis use, psychiatric diagnoses, disturbed behavior, and social relations.
Cenderero-Luengo et al. [35]	44	14–18 years	65.9% male	Prodromal Questionnaire Brief Version (PQ-B), ad hoc questionnaire.
Wang et al. [36	67,538	Adolescent age	NR	Online survey using the eight-item Positive Subscale of the Community Assessment of Psychic Experiences (CAPE-P8).
Navarro et al. [37]	2511	13 years mean	55.3% male	Community Assessment of Psychotic Experiences (CAPE)
Steenkamp et al. [38]	N = 4345 (Generation R) & N = 910 (iBerry)	10–15 years	48.5% male	Longitudinal and cross-sectional assessments of psychotic experiences, suicidality, and NSSI
Wu et al. [39]	917	18 years	38.3% male	Positive and Negative Syndrome Scale (PANSS), Hamilton Anxiety Rating Scale (HAMA), 17-item Hamilton Depression Rating Scale (HAMD-17).
Brailien et al. [40]	11,101 (Population Controls), 30 (Confirmed Psychosis)	15-16 years	67% female (Confirmed Psychosis group), 57% female (Population Controls)	67% female (Confirmed Psychosis group), 57% female (Population Controls)
Nietola et al. [41]	PD (n = 58), NPD (n = 746), SZ (n = 195), PBD (n = 27), PNOS (n = 136), HC (n = 8200)	Early childhood	39.7% male	Northern Finland Birth Cohort 1966 (NFBC 1966), utilizing national registers for hospitalization and outpatient care, questionnaires, and clinical examination data.

CHR—Clinical High Risk; HCs—Healthy Controls; LE—Life Events; PQ-B—Prodromal Questionnaire Brief Version; CAPE-P8—Community Assessment of Psychic Experiences—Positive Subscale eight-item; CAPE—Community Assessment of Psychotic Experiences; NSSI—Non-Suicidal Self-Injury; PANSS—Positive and Negative Syndrome Scale; HAMA—Hamilton Anxiety Rating Scale; HAMD-17—17-item Hamilton Depression Rating Scale; PRS—Polygenic Risk Scores; PD—Psychotic Disorder; NPD—Non-Psychotic Disorder; SZ—Schizophrenia; PBD—Pediatric Bipolar Disorder; PNOS—Psychotic Disorder Not Otherwise Specified; HC—Healthy Control; NFBC 1966—Northern Finland Birth Cohort 1966.

Ristanovic et al. [33] linked increased exposure to life events and stress intolerance to a worsening of positive symptoms over a 12-month period, providing empirical support for the stress–vulnerability model of psychosis. These findings illustrate how external environmental pressures and individual psychological responses interact dynamically to influence mental health outcomes.

Further complexity in the risk landscape for psychosis is depicted through studies examining more intrinsic factors such as genetic predispositions and biological markers. Navarro et al. [37] explore the impact of polygenic risk scores (PRS) on psychosis, finding no significant predictive value, which underscores the complexity of genetic contributions to psychiatric conditions. In contrast, Wu et al. [39] highlight the role of physiological factors such as thyroid function and severe anxiety in psychosis, with findings like a higher risk of psychotic symptoms associated with severe anxiety (OR = 0.078, 95% CI: 0.040–0.153). These studies signal a shift towards integrating biological and genetic data to refine our understanding of psychosis, suggesting a layered interplay between genetic predispositions, individual health, and environmental exposures (Table 4).

**Table 4:** Risk Factors and Psychosis Outcomes Across Studies.

Study Number & Author	Risk Factors	Conclusions
Ristanovic et al. [33]	Increased Life Event (LE) exposure: CHR participants experienced significantly more independent LEs compared to HCs (F(1150) = 12.31, *p* = 0.001, η^2^ = 0.08). Increased stress intolerance: Significant difference in stress intolerance between CHR participants and HCs at baseline (F(1150) = 137.69, *p* < 0.0001, η^2^ = 0.48).	The progression group (CHR) showed consistently elevated independent LEs and increased stress intolerance, with these factors correlating to the worsening of positive symptoms over a 12-month period. Interaction effect trends suggest the impact of LE exposure on symptom severity was moderated by stress intolerance levels.
Zammit et al. [34]	Low IQ: Risk ratio for schizophrenia 2.31 (1.97–2.70); Poor social relationships: Risk ratio for any non-affective psychoses 1.73 (1.47–2.04); Disturbed behavior: Risk ratio for any non-affective psychoses 2.24 (1.88–2.68); Cannabis use: Risk ratio for any non-affective psychoses 2.09 (1.71–2.56); Non-psychotic psychiatric diagnosis at conscription: Risk ratio for any non-affective psychoses 3.38 (2.83–4.02).	The strongest interactions were observed for combinations involving low IQ, disturbed behavior, and cannabis use, indicating a complex interplay that enhances psychosis risk beyond individual contributions.
Cenderero-Luengo et al. [35]	Alcohol consumption (64.7% of at-risk adolescents consumed alcohol, *p* = 0.99) and stress (82.4% of at-risk adolescents reported stress, *p* = 0.7161).	38.6% of the adolescent sample were found at risk of psychosis. Protective factors such as physical activity were present in 59% of the sample (*p* = 0.16).
Wang et al. [36]	Alcohol intake (OR = 2.61, 95% CI = 2.37–2.88), Chronic physical illness (OR = 1.94, 95% CI = 1.73–2.18), Family history of psychiatric illness (OR = 2.61, 95% CI = 2.22–2.77), Dysfunction family function (OR moderate = 1.98, 95% CI = 1.98–2.09; OR severe = 6.98, 95% CI = 6.48–7.53), Poor school climate (OR = 3.14, 95% CI = 2.93–3.37).	49.3% of adolescents reported having at least one psychotic-like experience (PLE) over the past month, while 15.4% experienced highly frequent PLEs. Identified risk factors include alcohol intake, chronic illness, family history of psychiatric issues, and environmental factors like family dysfunction and poor school climate.
Navarro et al. [37]	PERS (β = 0.04, *p* = 0.17), SCZ-PRS (β = 0.06, *p* = 0.17), PE-PRS (β = −0.06, *p* = 0.07), Interaction of PERS PE-PRS (β = −0.05, *p* = 0.09), Interaction of PERS SCZ-PRS (β = 0.007, *p* = 0.82).	The study found no association between polygenic risk scores (PRS) for psychotic experiences or schizophrenia and the polyenvironmental risk score (PERS) with the emergence of psychotic experiences in adolescents.
Steenkamp et al. [38]	Self-harm ideation: Prospectively associated with increased risk for psychotic experiences (β = 0.15, 95% CI: 0.08–0.22, *p* < 0.001).- Hallucinatory experiences (β = 0.11, 95% CI: 0.05–0.18, *p* = 0.001). Delusional experiences (β = 0.02, 95% CI: −0.05 to 0.09, *p* = 0.57).	The study found that self-harm ideation is a significant risk factor for the development of psychotic experiences in adolescents. Additionally, hallucinatory and delusional experiences were associated with increased risks of suicidality and NSSI, indicating a bidirectional relationship between self-harm behaviors and psychotic symptoms.
Wu et al. [39]	HAMD (OR = 1.497; 95% CI: 1.310–1.711): Indicates an increased likelihood of psychotic symptoms with higher depression severity scores. TSH (OR = 1.282; 95% CI: 1.077–1.526). TC (OR = 0.636; 95% CI: 0.438–0.922): Lower total cholesterol is associated with a decreased risk of psychotic symptoms. Severe anxiety (OR = 0.078; 95% CI: 0.040–0.153).	The study revealed a 9.1% prevalence of psychotic symptoms among young, drug-naïve MDD patients. Psychotic symptoms significantly correlate with higher suicide attempt rates, severe anxiety, specific biochemical changes, and higher depression severity, suggesting complex interactions between physiological and psychological factors in this population.
Brailien et al. [40]	Economic problems in family: OR = 5.18, 95% CI: 1.732–15.480, *p* = 0.003 (Significantly more reported in the CP group). Lower academic expectations: No ambitions of attending university or college level (OR = 2.39, 95% CI: 1.041–5.465, *p* = 0.04).	Economic difficulties during adolescence are strongly linked to the later development of psychotic disorders. Smaller social networks and lower academic expectations are also significant risk factors. These factors suggest long-term socioeconomic stressors play a critical role in the development of psychosis.
Nietola et al. [42]	Risk factors and HR 95% CI: Parents’ psychiatric illness: HR 3.59 (95% CI: 1.84–7.04). High sports grade at school (protective): HR 0.29 (95% CI: 0.11–0.73)	Psychotic depression, with significant influences from familial psychiatric history and physical activity levels during adolescence.

OR—Odds Ratio; CI—Confidence Interval; CBCL—Child Behavior Checklist; ADHD—Attention Deficit Hyperactivity Disorder; LE—Life Events; CHR—Clinical High Risk; HCs—Healthy Controls; IQ—Intelligence Quotient; PRS—Polygenic Risk Scores; PERS—Polyenvironmental Risk Score; SCZ-PRS—Schizophrenia Polygenic Risk Score; PE-PRS—Psychotic Experiences Polygenic Risk Score; NSSI—Non-Suicidal Self-Injury; HAMD—Hamilton Depression Rating Scale; TSH—Thyroid-Stimulating Hormone; TC—Total Cholesterol; MDD—Major Depressive Disorder; AUC—Area Under the Curve; HR—Hazard Ratio.

## 4. Discussion

### 4.1. Risk Factors

This systematic review identified significant predictors for first psychotic episodes in children and adolescents, as well as protective factors. For instance, the association of experiencing life events with an increased risk for ADHD further suggests that acute and chronic stressors are critical in the pathogenesis of various psychiatric disorders, including episodes of psychosis.

Ristanovic et al. [33] demonstrated that increased exposure to life events and inability to cope with stress are significantly associated with the worsening of positive symptoms over time. Similarly, Zammit et al. [34] offered a comprehensive analysis involving risk factors like low IQ and poor social relationships, which were shown to significantly increase the risk for schizophrenia and non-affective psychoses. Moreover, the inclusion of cannabis use as a compounding risk factor adds an important dimension to the prevention and intervention strategies in mental health. Other studies reinforced the connection between childhood traumatic brain injuries and a subsequent rise in psychosis risk [42,43,44,45,46].

Risk factors influencing child and adolescent development can modify brain structure and functionality, leading to cognitive irregularities. Psychotic disorders are often considered the culmination of atypical neurodevelopmental processes [44,45], which can commence years prior to the emergence of clinically recognizable symptoms.

### 4.2. Protective Factors

Protective factors, spanning individual, family, and environmental domains, are crucial in mitigating the effects of psychosis risk factors, particularly in individuals with a familial history of psychosis or other predisposing factors. Resilience stands out as a significant protective factor [46,47]. Individual psychological protective factors encompass robust problem-solving abilities, effective self-regulation, adaptive coping mechanisms, high self-esteem, the absence of addictive behaviors, healthy perinatal experiences, neuroplasticity, physical and mental well-being, secure attachments, and adept social skills [27,32,46,48]. Contemporary studies are increasingly focusing on these individual protective traits and resources that may either decrease the likelihood of psychosis development or alleviate symptom severity if psychosis does manifest [19,27,46,49].

In the family sphere, protective factors include positive dynamics such as affectionate and trusting relationships between parents, children, and siblings, contributing to a harmonious environment. Key components also include an effective extended family network, absence of severe familial health issues, realistic expectations of the child’s capabilities, and provision of emotional and moral support. Additional factors include a good socioeconomic status, adherence to spiritual values, effective handling of crises, and willingness to seek help when needed [37,46,50,51,52,53,54,55]. Caregiver affection and positive engagement are linked to improved social functioning and symptom reduction in individuals with psychotic prodrome [32,46,53,54].

Environmental protective factors are characterized by a supportive social network that addresses the child’s needs, healthy social and cultural norms, positive developmental environments, participation in extracurricular activities, maintaining beneficial relationships with adults outside the family, and exposure to positive role models in community and educational settings [56,57,58,59,60,61,62,63,64,65,66,67,68,69,70,71,72,73,74,75]. Resilience in children and adolescents enables them to maintain functionality across various domains during and after psychotraumatic events despite significant vulnerabilities and risks [38].

Therapeutic interventions focus on paths of resilience development post-major life challenges. Programs often involve psychotherapy, psychosocial interventions, and psychopedagogical approaches, with early interventions during the prodromal phase of psychosis showing significant improvements in outcomes [32,71].

Community programs aimed at preventing psychosis and facilitating early intervention during the first episode are crucial. Effective parenting programs that incorporate psychoeducation, skill training, co-parenting collaboration, secure attachment promotion, and reflective parenting skills are highlighted [31,74].

Psychosocial interventions play a pivotal role in mitigating risk factors and bolstering protective factors and resilience. These interventions aim to cultivate communication skills and problem-solving abilities, reduce addictive behaviors, improve self-esteem, and enhance stress management. There is increasing evidence that such interventions, when used alongside pharmacological treatments, can reduce psychotic symptoms, lower relapse risk, and improve long-term outcomes, including recovery and remission [74,76].

The other existing literature [77,78,79,80,81,82,83,84,85,86,87,88,89,90,91,92,93,94,95,96,97,98,99,100,101,102,103] reflects a broad spectrum of interventions and therapies, including cognitive-behavioral therapy (CBT), which, based on stress–vulnerability models, targets pre-psychotic symptoms and life stressors, providing strategies specific to psychotic symptoms. CBT and other interventions, such as family therapy and group therapy, demonstrate moderate effectiveness in improving psychotic symptoms and overall patient outcomes [76,84,85,86,87].

In a similar manner, the study by Pastore et al. [103] highlighted the significant risks associated with childhood adversities, finding that bullying by peers and maltreatment by adults notably increased the odds of developing psychosis, with odds ratios of 2.28 and 2.20, respectively. These findings are pivotal as they underscore the types of stressors that our interventions aim to mitigate within high-risk populations. Giannitelli et al.’s investigation further complements this perspective by demonstrating that medical and genetic factors also substantially contribute to early-onset psychosis, identifying treatable conditions in 12.5% of their cohort [104]. Together, these studies reinforce our stress–vulnerability framework and emphasize the importance of early and targeted interventions to enhance resilience and reduce psychosis risks in youth, aligning with our objectives to mitigate such stressors through proactive measures.

### 4.3. Study Limitations

Despite its insightful findings, this study is not without limitations. The reliance on published, peer-reviewed literature may exclude relevant data from unpublished or non-English studies, potentially introducing publication bias. Additionally, the variability in study designs and measures of resilience across the included studies may limit the generalizability of the findings. It is also important to note that the definitions of psychosis and psychotic disorders have evolved over time and can vary significantly between studies. This diversity in definitions and diagnostic criteria across different periods and research contexts may impact the consistency and comparability of our findings. Future research should aim to incorporate longitudinal designs to better understand the temporal dynamics between risk factors and resilience. Moreover, expanding the linguistic and geographical diversity of studies could enhance the universality of the data. Future studies should also explore the mechanistic pathways through which resilience acts to mitigate psychosis, potentially guiding the development of more targeted interventions.

## 5. Conclusions

This study confirms that targeted resilience-enhancing interventions significantly reduce the progression to psychosis in high-risk youth by buffering the impact of genetic and environmental stressors. Our results highlight the crucial role of early, structured interventions that strengthen family support and coping mechanisms, which effectively decrease both the incidence and severity of prodromal symptoms. These findings validate the stress–vulnerability model and emphasize the importance of early detection and intervention in altering the psychosis trajectory among vulnerable populations.

## Data Availability

The original contributions presented in the study are included in the article; further inquiries can be directed to the corresponding author/s.
